# A50 THE EFFECTS OF SHORT-CHAIN FATTY ACIDS ON EPITHELIAL AND MACROPHAGE RESPONSE TO BACTEROIDES FRAGILIS

**DOI:** 10.1093/jcag/gwac036.050

**Published:** 2023-03-07

**Authors:** M Bording-Jorgensen, K Chea, T Gartke, C Cheng, H Armstrong, E Wine

**Affiliations:** 1 University of Alberta, Edmonton; 2 University of Manitoba, Winnipeg, Canada

## Abstract

**Background:**

Patients with IBD have an altered microbiome and gut microenvironment leading to changes in their Short-Chain Fatty Acid (SCFA) profile. Pathobionts are commensal organisms that become pathogenic under specific conditions, likely related to microenvironmental gut changes. This is especially relevant to children given that early life exposures are critical to microbiome development and impact immune response.

**Purpose:**

The objectives of our study were to: 1) determine if microbes from IBD patients are more proinflammatory compared to non-IBD microbes; and 2) define how SCFAs affect host-response to potential pathobionts.

**Method:**

Fructooligosaccharide (FOS) fermentation and SCFA production were assessed by HPLC-RID using whole-intestinal microbe culture collected as mucosal washes from pIBD (n=10) and non-IBD patients (n=8). Individual anaerobic bacteria were isolated from individual patients and screened for proinflammatory response [interleukin (IL)-1ß] using THP-1 macrophages. Caco-2 (epithelial) or THP-1 cells were pre-exposed to individual SCFAs (50 mM acetate or formate; 10 mM succinate; or 1 mM butyrate or propionate), then infected with *Bacteroides fragilis*, isolated from patients. Microscopy (bacterial staining with HEMA 3), qPCR, ELISA, TransEpithelial Electrical Resistance (TEER), and culturing were used to determine invasion potential, cytokine expression, barrier, and secretion.

**Result(s):**

FOS fermentation by microbes from IBD patients led to increased acetate and decreased butyrate production. Microbes from IBD patients were more proinflammatory, with increased IL-1ß secretion and reactive oxygen species generation, compared to those from non-IBD patients. *B. fragilis* isolated from an IBD patient was found to be a potential pathobiont with increased invasion and cytokine production in both epithelial and macrophage cells. Invasion was further increased when cells were exposed to SCFAs, particularly acetate and butyrate. Furthermore, the strain isolated from the IBD patient was observed (by microscopy) to be more adherent to the epithelial barrier, causing a loss of membrane integrity (TEER). The inflammasome pathway were significantly upregulated in Caco-2 cells infected with IBD-isolated *B. fragilis* and incubated with acetate or butyrate.

**Conclusion(s):**

The altered microbiome in IBD patients leads to an increased acetate and decreased butyrate, which likely affects host response to microbes and promotes inflammation. This changed microenvironment promotes the development of pathobionts, such as the identified *B. fragilis* strain we isolated from an IBD patient. The observation that acetate and butyrate increased host susceptibility to this strain indicates that understanding diet and SCFA production are instrumental in treating chronic conditions such as IBD. The role of diet as both a treatment and potential cause of inflammation in IBD is becoming more apparent; therefore, it is crucial that we understand the complex role diet has in host-microbe interactions.

**Please acknowledge all funding agencies by checking the applicable boxes below:**

CIHR, Other

**Please indicate your source of funding;:**

Mitacs- Weston Foundation

**Disclosure of Interest:**

None Declared

